# Pethidine in Low Doses versus Dipyrone for Pain Relief in Labor: A Randomized Controlled Trial

**DOI:** 10.1055/s-0038-1676509

**Published:** 2019-02

**Authors:** Rogevando Rodrigues Nunes, André Montenegro Primo

**Affiliations:** 1 Graduate Program in Medical Sciences, Universidade de Fortaleza, Fortaleza, CE, Brazil

**Keywords:** analgesia, labor, dipyrone, pethidine, humanizing delivery, analgesia, trabalho de parto, dipirona, petidina, parto humanizado

## Abstract

**Objective** To compare low doses of pethidine with dipyrone in labor analgesia.

**Methods** In a randomized prospective study conducted by Universidade de Fortaleza, in the state of Ceará, Brazil, between May and December 2016, 200 full-term parturients, with very painful uterine contractions and exhibiting uterine cervix dilatation ≥ 5 cm, were selected to receive a single intravenous dose of either 0.25 mg/kg of pethidine (*n* = 100) or of 25 mg/kg of dipyrone (*n* = 100). Pain was assessed using the visual analogue scale. The data were analyzed using the Student *t*-test, the chi-square test and the likelihood ratio.

**Results** There was a significant improvement in pain in 35% of the parturients. Both drugs presented a similar analgesic effect 1 hour after the intervention (*p* = 0.692). There was no analgesic effect during the evaluation of the second hour after the intervention with pethidine or dipyrone. There were no adverse effects, such as maternal drowsiness, nausea or vomiting, related to the drugs used.

**Conclusion** Pethidine in low doses and dipyrone presented equivalent analgesia during labor.

**Public Registry of Clinical Trials** RBR-4hsyy4.

## Abstract (pt)

**Resumo:**

**Objetivo** Comparar doses baixas de petidina com dipirona na analgesia de parto.

**Métodos** Em um estudo prospectivo randomizado realizado pela Universidade de Fortaleza, Ceará, Brasil, entre maio e dezembro de 2016, 200 parturientes a termo, com contrações uterinas muito dolorosas e apresentando dilatação do colo uterino ≥ 5 cm, foram selecionadas para receber dose única intravenosa de 0,25 mg/kg de petidina (*n* = 100) ou 25 mg/kg de dipirona (*n* = 100). A dor foi avaliada pela escala visual analógica. Os dados foram analisados por meio dos testes *t* de Student, qui-quadrado e razão de verossimilhança.

**Resultados** Houve melhora significativa da dor em 35% das parturientes. Ambas as drogas apresentaram efeito analgésico semelhante 1 hora após a intervenção (*p* = 0.692). Inexistiu efeito analgésico durante a avaliação da segunda hora após a intervenção com a petidina ou com a dipirona. Não houve efeitos adversos, como sonolência, náuseas ou vômitos maternos, relacionados aos medicamentos utilizados.

**Conclusão** A petidina em doses baixas e a dipirona apresentaram analgesia equivalente durante o trabalho de parto.

**Registro público de testes clínicos** RBR-4hsyy4.

## Introduction

Pain control has always been a major concern of mankind, although it is often neglected and trivialized. In ancient times (prior to 476 A.D.), pain was believed to be a form of punishment from the gods or the demons.

Throughout the twentieth century, several drugs such as aspirin, pethidine, dipyrone, opium and ergotamine began to be employed to control pain. After that, more and more powerful analgesics began to emerge.^1^

Women often describe labor pain as the most intense painful sensation they have ever experienced. When the pain is very strong, it can lead to psychological trauma and, in some cases, negatively interfere with the normal course of labor.^2^ Posttraumatic stress disorder is a condition that is observed in 1 to 2% of postpartum patients.^3^

The high rates of cesarean births practiced in Brazil in recent years reach 55.6%, contrasting with the 15% recommended by the World Health Organization (WHO).^4^ These high percentages reflect factors such as the phobia pregnant women feel regarding the pain of labor.

These factors determine a great need to find ways to relieve pain in these women.

Continuous epidural, which is used since 1947, is currently considered the gold standard in labor analgesia.^5^ However, it requires relevant additional financial resources, which hampers its use on a large scale. In countries where health investments are relatively scarce, such as Brazil, less costly analgesic options are required.

Pethidine has been used in labor since 1940. Well-documented studies employing dipyrone for this purpose have not been conducted. Both drugs are relatively inexpensive, easy to handle and safe, when used sparingly. Doses of up to 50 mg of intravenous (IV) or intramuscular (IM) pethidine do not cause relevant changes in maternal-fetal vitality.^6^ ^7^ ^8^ ^9^ ^10^ ^11^

Most studies found in the literature employ fixed doses of IV or IM pethidine ranging from 25 mg to 100 mg during labor.^12^ ^13^ ^14^ ^15^ ^16^ After a search performed within the Capes, PubMed/Medline and BVS databases, from January 1st, 2000, to February 9th, 2016, we did not identify published research articles using doses of up to 50 mg of IV pethidine during labor in which the dosages were individualized according to the body mass of the parturients.

Individual body mass may exert a strong influence on the effect of a given drug. In view of the inconvenience of routinely obtaining the blood concentration of drugs, it is more practical to administer them taking into consideration the individual body mass.

During pharmacological analgesia, it is necessary to administer an adequate dosage of the drug so that the pregnant woman does not manifest unwanted effects, and to maintain the vitality of the newborns.

This study compared the effects of pethidine (at low doses) and sodium dipyrone, when used in labor analgesia. Moreover, the Apgar scores were analyzed in the first and fifth minutes of life of the newborns, as well as the percentage of newborns who needed supplemental oxygen for a period > 30 minutes during the first hour of life.

## Methods

We conducted a controlled clinical trial. The study protocol was previously approved by Ethics in Research Committee of Universidade de Fortaleza (under CAAE 53754015.0.0000.5052). Informed consent was obtained previously from all of the parturients in the study.

Recruitment of the study sample took place through the distribution of posters at the hospital where the research was performed. A controlled, double-blinded trial randomized in 20 blocks was conducted. The research took place in Hospital Nossa Senhora da Conceição (HNSC), which is part of the hospital structure of the city of Fortaleza, in the state of Ceará, Brazil. The mentioned hospital does not have a labor analgesia service. The research was performed from May to December 2016.

The sample population (n) was defined using the variance, the significance, the minimum difference between the mean scores attributed to pain that could be identified in both groups, and the statistical power described in previous similar studies.^17^ ^18^ The study power was of 90%, with a significance of 5% (α = 0.05, β = 0.1).

The present study recruited 200 parturients, and half (*n* = 100) of them were given 0.25 mg/kg of pethidine IV, and the other half (*n* = 100) were given 25 mg/kg of dipyrone IV, in a single dose. The drugs were diluted in 18 ml of distilled water and administered slowly, over the course of 5 minutes. The parturients received the analgesic as soon as they met the criteria required for admissibility in the research. During the research, one assistant generated the sequence of interventions on the computer, another collaborator prepared the medications, and another assistant administered the painkiller. The researcher collected the data from each participant. Neither the researcher nor the participants knew which of the two drugs had been administered to whom. The data were analyzed using the Predictive Analytic Software for Windows (PASW, SPSS Inc., Chicago, Il, US), version 17. The unpaired Student *t*-test, the same test paired with Bonferroni correction, the chi-squared (χ^2^) test and the likelihood ratio, whenever appropriate, were used. The quantitative analysis was performed using the Student *t*-test. For the qualitative analysis, the likelihood ratio and the χ^2^ test were used.

The pain levels were assessed using the visual analogue scale (VAS), which has been an internationally recognized method for this purpose since 1976.^19^ The VAS scores the pain from “0” to “10,” with “0” being the absence of pain and “10” being the worst pain the participant believes she can endure. The pain was measured just before the analgesics were applied, as well as 1 hour and 2 hours later. This measurement was performed 1 minute after the end of the last painful uterine contraction. The medication was administered only to the parturients who reported intense pain resulting from uterine activity, with scores of 8, 9 or 10 on the VAS.

During the hospitalization, the study was explained to the parturients. They received information about the fact that they were volunteering for the study, the probable benefits of the medications over pain, and the possible side effects they might experience, such as nausea, vomiting, allergic reactions, pain due to the venous puncture etc.

The inclusion criteria, which were always concomitantly present, were: a) presence of ≥ 3 uterine contractions lasting ≥ 50 seconds every 10 minutes; b) parturients presenting a dilation of the uterine cervix ≥ 5 cm; c) intense pain; d) gestational age ≥ 37 weeks and ≤ 40 weeks and 6 days; e) cephalic presentation of the fetuses.

The exclusion criteria were: a) parturients with any of the following events: poorly-controlled arterial hypertension (with hypertensive spikes), diabetes, restricted intrauterine growth and placental abruption; b) use of oxytocin in labor; c) previously diagnosed and untreated hypothyroidism; d) users of monoamine oxidase inhibitors; e) the birth occurring in less than 1 hour after the intervention; f) parturients with body mass ≥ 100 kg; g) indication of cesarean delivery during admission; h) previously-diagnosed Addison disease; i) fetal heart rate (FHR) < 110 bpm or > 160 bpm upon admission; j) patients allergic to sodium dipyrone; k) patients allergic to pethidine; l) chlorpromazine users; m) phenobarbital users; n) phenytoin users; o) fetuses without vitality; p) twin pregnancy; q) drug addiction.

**Fig. 1:**
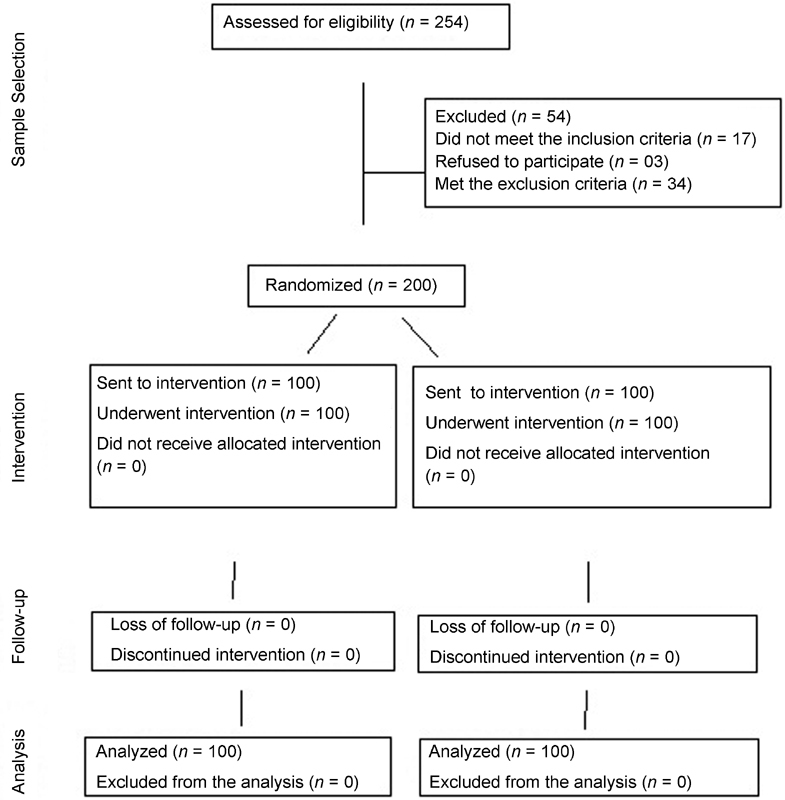
Flow chart of the participants throughout the study.

Initially, 254 parturients were selected. After the inclusion and exclusion criteria were applied, only 200 parturients met all of the requirements and became participants of the study (Fig. 1).

## Results

In the evaluation performed 1 hour after drug administration, an improvement in pain in the 2 groups was verified, benefiting 35% of the parturients. The primiparas represented 46.5% (93) of the sample; 44 of them used pethidine, and 49 used dipyrone. The mean age of the participants was 23.94 years for both groups, ranging from 14 to 44 years. During the assessment performed two hours after the intervention, no analgesic effect was described in the two groups. During the research, the drugs presented equivalent analgesic effects (Table 1).

**Table 1:** Pain during the course of labor using the Visual Analogue Scale

Time when the pain was evaluated	Dipyrone (*n* = 100)	Pethidine (*n* = 100)	*p*-value^a^
Preanalgesia	8.47 ± 0.559	8.55 ± 0.626	0.341
Preanalgesia/1 hour postanalgesia	8.47 ± 0.559/7.97 ± 1.226	–	< 0.001^b^
Preanalgesia/1 hour postanalgesia	–	8.55 ± 0.626/8.04 ± 1.271	< 0.001^b^
Preanalgesia/2 hours postanalgesia	8.38 ± 0.58/8.36 ± 1.00	–	0.908
Preanalgesia/2 hours postanalgesia	–	8.44 ± 0.56/8.52 ± 1.04	0.497
1 hour postanalgesia	7.97 ± 1.226	8.04 ± 1.271	0.692
2 hour postanalgesia	8.36 ± 1.002	8.52 ± 1.039	0.397

Notes: ^a^Student *t*-test; ^b^statistically significant difference (*p*-value < 0.05).

The Apgar scores at 1 and 5 minutes of life of the newborns did not show statistically significant differences during the comparison between the groups (Table 2).

**Table 2:** Apgar score for the newborns of mothers who underwent labor analgesia

Variables	Dipyrone (*n* = 100)	Pethidine (*n* = 100)	*p*-value^a^
Apgar 1 minute	8.08 ± 0.950	8.09 ± 0.570	0.928
Apgar 5 minutes	8.90 ± 0.541	9.00 ± 0.142	0.077

Note: ^a^Student *t*-test; statistically significant difference (*p*-value < 0.05).

The use of supplemental oxygen by the newborns over a period of more than 30 minutes during the first hour of life did not result in statistically significant differences between the groups (Table 3).

**Table 3:** Use of supplemental oxygen by the newborns of the parturients submitted to labor analgesia

Variables	Dipyrone (*n* = 100)	Pethidine (*n* = 100)	*p-*value^a^
Use of supplemental oxygen for > 30 minutes during the first hour of life	6 (6%)	9 (9%)	0.421

Note: ^a^Chi-squared test or likelihood ratio; statistically significant difference (*p*-value < 0.05).

There were no adverse effects, such as maternal drowsiness, nausea or vomiting, related to the drugs used.

## Discussion

The pain relief obtained during the assessment performed 1 hour after analgesic administration was significant. The lowest score attributed to pain on the VAS was 7.97. The analgesia provided by both pethidine and dipyrone lost its effect after 2 hours. The Apgar score did not vary between the groups. The percentage of newborns that used supplemental oxygen for more than 30 minutes during the first hour of life was similar in both groups.

In a research using 50 mg of pethidine IV, theVAS pain scores observed were of 6.6 and 7.3 respectively, at 1 hour and 2 hours after the administration of analgesics.^8^ A similar result was described in the study conducted by Khooshideh and Shahriari^6^ 1 hour after the administration of 50 mg of IM pethidine (VAS = 7). These results are consistent with the present research, because, using a mean dose of IV pethidine of 16 to 20 mg (0.25 mg/kg) both the duration and the intensity of the analgesia were lower than with the administration of 50 mg.

A study^7^ conducted in Egypt using 50 mg of IV pethidine, and another^10^ in Iran, which used 50 mg of IM pethidine, did not observe differences in the Apgar scores at 1 and 5 minutes when compared with placebo.

A research^20^ published in Uruguay used 100 mg of IV pethidine in the parturients and observed respiratory depression and acidosis in the newborns. Another study^21^ employed 100 mg of IM pethidine every 3 hours during labor, and reported that 29.4% of the infants required nursery admission for special care.

The limitation of this study is due to the fact that the painful sensation related to labor assumes a magnitude related to the culture of each population. Due to this detail, the results found may not be reproducible in populations that are very different from our sample.

Some suggestions for the development of future researches involving these two drugs are pertinent, as follows:

1. Considering that the two analgesics have different mechanisms of action and cause significant effects in the first hour after administration, increases in the intensity of the analgesia and in the period of action, if the both drugs are used simultaneously, are expected.
2. Administration of only one of the two drugs. The doses would be increased to: the 35 mg/kg of dipyrone would be increased to up to 2.5 g, and the 0.5 mg/kg of pethidine, up to 50 mg, to try to achieve a more intense and longer relief of the painful sensation. This would be a more adequate suggestion for intense pain, such as in cases of VAS scores of 8.
3. Administration of the 2 drugs with increased dosages according to item 2, simultaneously, when the birth is expected to occur in the next 2 hours. The amount of benefited parturients should be greater than when employing merely one drug, and a more intense and longer lasting analgesia is presumed to occur. This option seems more appropriate for levels of intense pain, in which VAS = 9 or 10.
4. Administration of the 2 drugs, using increased dosages according to item 2, interspersed by 1.5 hour to 2 hours, if the initial prediction is for birth after 2 hours of the intervention. The intention is to achieve a longer-lasting, but less pronounced analgesic effect than the simultaneous administration of the drugs.

It is expected that, when labor analgesia becomes more accessible to the population, many women who are prone to having their child through surgery will opt for normal delivery. The health system itself would be favored by the reduction in cesarean sections, which would result in fewer infections and lower expenses, both on the part the state and on the part of the patients.

During labor, low doses of pethidine and dipyrone displayed similar analgesic effects measured at 1 hour after the IV administration of the drugs. However, the analgesic effect was not noticed during the evaluation conducted 2 hours after the intervention, regarding both drugs.

As for the newborns, no differences in the Apgar scores at 1 and 5 minutes were observed, and the use of supplemental oxygen for a period exceeding 30 minutes during the first hour postpartum was similar in both groups.

The current recommendation of the WHO is that the treatment of severe labor pain should be performed initially through non-pharmacological methods. The main non-pharmacological methods used in labor analgesia are: acupuncture, massages, ambulation, breathing exercises, shower baths, immersion baths, and cryotherapy (ice packs applied to the sacral region), as well as psychological methods such as audioanalgesia, hypnosis and presence of a companion for the patient in the delivery room. The pharmacological methods should be reserved if there is absence or ineffectiveness of the non-pharmacological methods.^22^ ^23^ ^24^

## Conclusion

During labor, pethidine at low doses (0.25 mg/kg) and dipyrone at usual doses (25 mg/kg) presented equivalent analgesic effects 1 hour and 2 hours postintervention. The analgesic effect, however, lasted only during the first hour of evaluation for both drugs. The occurrence of adverse events for mothers and concepts was similar and indicative that both analgesia plans are safe during labor.
